# The segregation of organelles and organellar genomes across eukaryotic biology

**DOI:** 10.1042/BST20253120

**Published:** 2026-03-13

**Authors:** Clirim Jetishi, Markus Gerber, Torsten Ochsenreiter

**Affiliations:** 1 Institute of Cell Biology, University of Bern, Bern 3012, Switzerland; 2 Graduate School for Cellular and Biomedical Sciences, University of Bern, Bern 3012, Switzerland

**Keywords:** apicoplast, chloroplast, DNA segregation, membrane tethering, mitochondrion, mitosomes, organellar DNA, tripartite attachment complex

## Abstract

Eukaryotic cells are characterized by the presence of organelles such as mitochondria and, in the case of plants and certain protists, plastids, both of which often contain their own genomes. Accurate distribution of replicated organelles and their genomes to daughter cells is crucial for cell survival and propagation across all eukaryotic organisms. Unlike nuclear DNA, which follows a well-characterized segregation process via the mitotic spindle, organelle genomes are inherited through more diverse and less-understood mechanisms. Ensuring proper organelle genome inheritance is essential for maintaining cellular energy production, metabolic functions, and overall viability. Because organelle and organelle genome segregation lack a universal mechanism, different organisms employ various strategies that include stochastic distribution and active cytoskeletal transport and membrane tethering to prevent the loss of essential genetic material while supporting organelle division and turnover. This review provides an overview of organelle and organellar DNA segregation mechanisms in diverse eukaryotic systems before focusing on the tripartite attachment complex as a specialized adaptation in kinetoplastid parasites.

## Plastids

### Plants

**Figure 1:**
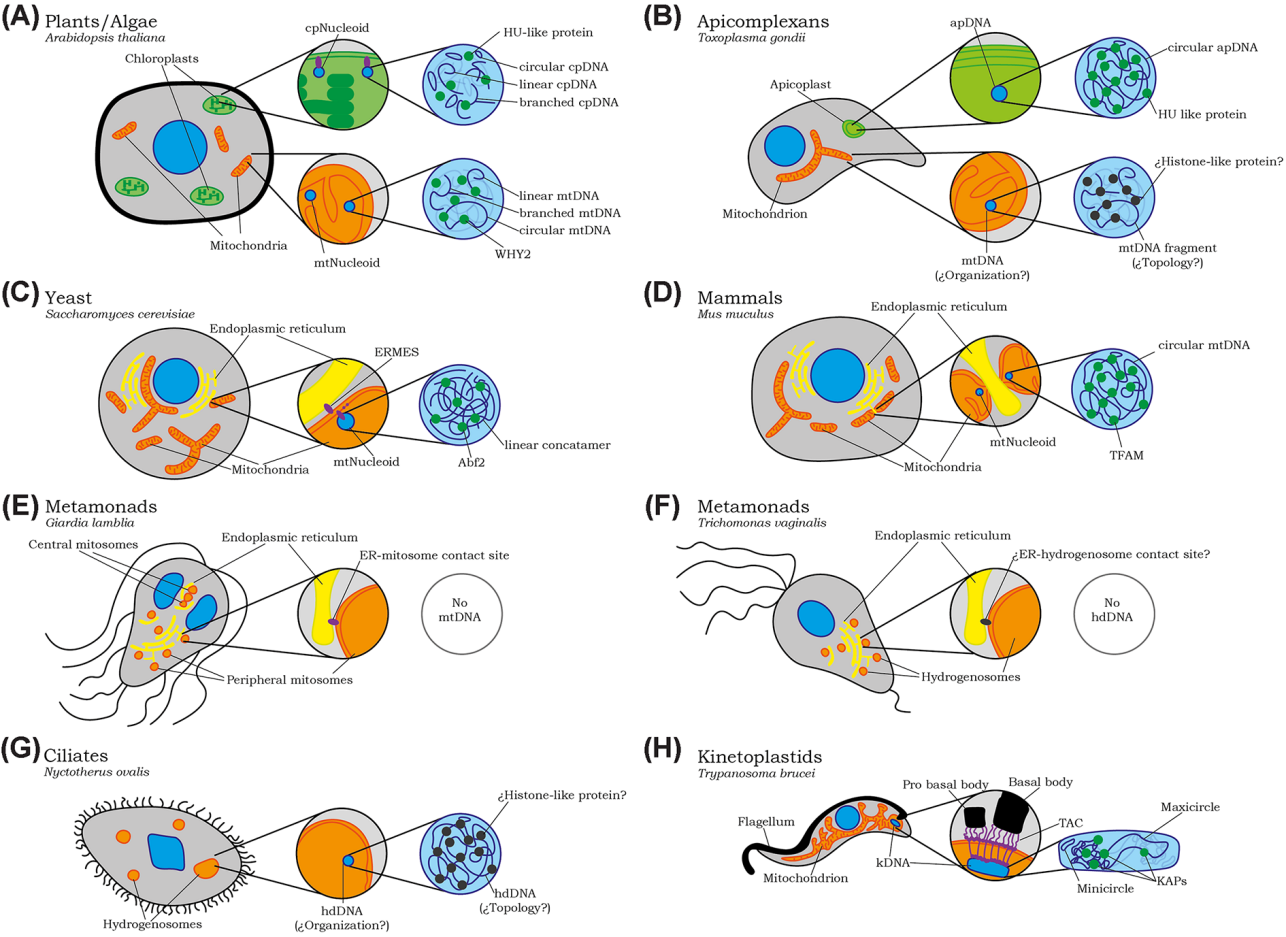
Diversity of genome organization in plastids, mitochondria, and mitochondria-related organelles across eukaryotes Schematic overview of mitochondrial, plastid, and mitochondria-related organelle (MRO) genome organization and their association with the endoplasmic reticulum (ER) or the flagellum in representative eukaryotic lineages. (**A**) Plants/algae (*Arabidopsis thaliana*): Chloroplast nucleoids contain circular, linear, and branched cpDNA packaged by HU-like proteins. Plant mitochondrial nucleoids comprise heterogeneous mtDNA molecules of mixed topology associated with mitochondrial nucleoid proteins, including WHY2. (**B**) Apicomplexans (*Toxoplasma gondii*): The apicoplast harbors a circular plastid genome organized by HU-like proteins. The single mitochondrion contains a highly fragmented mtDNA genome; its topology and the identity of dedicated nucleoid-organizing proteins remain unknown. (**C**) Yeast (*Saccharomyces cerevisiae*): Mitochondrial DNA is present as linear concatemeric molecules packaged by the HMG-box protein Abf2. Mitochondria form defined contact sites with the ER via the ER-mitochondria encounter structure (ERMES). (**D**) Mammals (*Mus musculus*): Circular mtDNA molecules are organized into discrete nucleoids by TFAM and are closely associated with ER-mitochondria contact sites. (**E**) Metamonads (*Giardia intestinalis*): Genome-free mitosomes are constitutively associated with the ER through stable contact sites and lack mitochondrial DNA. (**F**) Metamonads (*Trichomonas vaginalis*): Hydrogenosomes lack DNA and are frequently observed in close association with the ER, although the molecular basis of these contacts remains unknown. (**G**) Ciliates (*Nyctotherus ovalis*): Hydrogenosomes retain a mitochondrial-derived genome (hdDNA); its organization, topology, and associated DNA-packaging proteins have not been identified. (**H**) Kinetoplastids (*Trypanosoma brucei*): The single mitochondrion contains the kinetoplast DNA (kDNA) network composed of interlocked minicircles and maxicircles, organized by kinetoplast-associated proteins (KAPs) and physically linked to the basal body via the tripartite attachment complex (TAC).

Plastids, [1–3] including chloroplasts in plants and algae, possess their own genomes (plastid DNA, ptDNA) organized into multiple copies packaged as nucleoids (Table 1 and Figure 1) [4,5]. Plastid genome sizes range from 120 to 190 kilobases (kb) in photosynthetically active algae and higher plants [6]. PtDNA exists as a heterogeneous mixture of monomers, concatemers, and complex branched structures [4]. Chloroplast nucleoids, consisting of DNA and associated proteins, are organized by HU-like DNA-binding proteins that are homologous to the bacterial histone-like protein HU and vary in shape, number, and distribution among species (Table 1) [4,5,7]. During chloroplast division, nucleoids are believed to anchor to the inner envelope membrane, helping to organize and evenly partition DNA between daughter plastids (Figure 1) [8].

Chloroplast division occurs by constriction, relying on mechanisms inherited from their cyanobacterial ancestors. The tubulin-like GTPase FtsZ assembles into a contractile Z-ring at the division site, guiding membrane constriction [9,10]. In addition to FtsZ, cytoplasmic dynamin-related proteins (DRPs) such as DRP5B (also known as ARC5) and the plastid-dividing (PD) ring assist in the final separation of chloroplasts [11,12]. During this process, nucleoids must be segregated properly. In *Chlamydomonas reinhardtii*, chloroplast nucleoids temporarily disperse into a network before division into four daughter chloroplasts [4]. Proteins like Monokaryotic Chloroplast 1, a Holliday junction resolvase, help untangle replicated ptDNA, promoting accurate distribution to daughter organelles [5]. Despite these discoveries, many aspects of chloroplast nucleoid segregation remain unresolved.

### Apicomplexan parasites and the apicoplast

Apicomplexan parasites, such as *Plasmodium* species and *T. gondii*, contain a non-photosynthetic plastid known as the apicoplast. Before replication, this organelle houses a small, spherical nucleoid that contains multiple circular genomes (∼35 kb) (Table 1 and Figure 1) [13,14]. This nucleoid is organized and compacted by the histone-like protein HU [15,16]. Because each parasite cell contains only one apicoplast, faithful segregation during division is critical [17]. To achieve this, the apicoplast physically connects to the centrosomes. In *T. gondii*, the apicoplast stretches into a U-shape during nuclear division, reaching into the two forming daughter cells—a process known as endodyogeny—with the newly replicated nucleoids positioned near the centrosomes. As daughter cells mature, the apicoplast remains attached to duplicated centrosomes until medial fission separates it into two organelles, each inherited by a daughter cell [18].

Apicoplast inheritance in *T. gondii* also requires an actomyosin-based mechanism. The class XXII unconventional myosin TgMyoF localizes near the apicoplast and is essential for segregation [19]. Disrupting actin dynamics or TgMyoF function results in parasites failing to inherit the apicoplast, which is lethal for the cell. Although TgMyoF is found near the apicoplast, its primary role seems to involve proper centrosome positioning, indirectly affecting apicoplast inheritance [19].

Apicoplast nucleoid partitioning begins early, at the onset of S-phase before centrosome duplication and spindle formation [20]. As the apicoplast elongates, the nucleoids segregate progressively toward the growing ends of the organelle. This coupling of nucleoid segregation and apicoplast elongation highlights a tightly coordinated process, although the precise molecular factors remain largely unknown.

## Mitochondria

### Opisthokonts

In opisthokonts, mitochondrial inheritance has been extensively studied in yeast and mammalian systems. In *Saccharomyces cerevisiae*, mitochondria form a dynamic tubular network containing multiple mitochondrial DNA (mtDNA) nucleoids distributed throughout (Table 1 and Figure 1) [21]. During asymmetric division by budding, mitochondria are actively transported into the daughter bud along actin cables by the class V myosin Myo2p, assisted by adaptor proteins and GTPases [22,23].

The *S. cerevisiae* mitochondrial genome primarily consists of linear molecules (∼75–150 kb), accompanied by smaller circular forms [22]. Each cell harbors 10–40 nucleoids, anchored to the inner mitochondrial membrane (IMM) and packaged by the HMG-box protein Abf2, which condenses and stabilizes the mtDNA [24,25]. Nucleoids in yeast are spaced approximately 800 nm apart, with preferential localization near mitochondrial tips, suggesting a tightly regulated distribution [26].

Mitochondrial inheritance in *S. cerevisiae* is further coordinated by contact sites between mitochondria and the ER, mediated by the ERMES complex [27]. This complex consists of four proteins (Mdm10, 12, 34, and Mmm1) and connects the mitochondrial outer membrane to the ER for lipid transfer (Table 1 and Figure 1) [28]. Furthermore, the complex links replicating nucleoids to future division sites and thus plays a crucial role in coordinating mitochondrial division and ensures accurate nucleoid segregation [29]. While mitochondrial fission and fusion were initially thought essential for mtDNA inheritance [30], cells lacking these processes still distribute nucleoids but accumulate defective genomes, indicating a role in mtDNA quality control rather than physical segregation [26,31].

Similarly, in mammals, mitochondrial inheritance relies on dynamic organization of the network. Mammalian cells contain numerous mitochondria, hosting hundreds to thousands of mtDNA copies organized into compact nucleoids typically containing a single genome (Table 1 and Figure 1) [32–34]. The mammalian nucleoid is about 100 nm in diameter with an average of 1.4 mt genomes per nucleoid, and more recent studies in mice have identified a set of twelve core nucleoid proteins comprising eight mtDNA replication/transcription factors and four mitoribosome-associated proteins [35,36]. Mitochondrial nucleoids are compacted and tethered to the IMM, a connection essential for their positioning, trafficking, and distribution within the network. However, the exact molecular mechanism of this tethering remains unknown, with multiple proteins, membrane lipids, and contact site structures proposed but not yet fully understood [37,38]. The human mitochondrial genome is a circular 16,569 bp DNA encoding 13 oxidative phosphorylation proteins, 22 tRNAs, and two rRNAs [39,40]. Mitochondrial transcription factor A (TFAM), the major packaging protein, wraps mtDNA into tight nucleoid structures while also regulating transcription and copy number [39,41].

Like yeast, mammalian mitochondria undergo ER-associated mitochondrial division (ERMD), where ER tubules encircle mitochondria to specify constriction sites and recruit highly conserved DRPs [29,42]. In both mammalian cells and yeast, the process of ERMD has been coupled to the actin cytoskeleton, suggesting that mitochondrial constriction during division might be mediated by actin. mtDNA replication often initiates at these ER-mitochondria contact points, and newly replicated genomes require mitochondrial topoisomerase TOP3α to resolve interlinked molecules before segregation [34,43]. Fission and fusion events, together with cytoskeletal transport involving kinesins, dyneins, and myosin XIX, distribute nucleoids evenly across the mitochondrial network and ensure their partitioning during cell division [44–46]. These integrated mechanisms preserve mtDNA inheritance and integrity across opisthokont species.

### Plants

Rather than forming extensive networks, plant mitochondria exist mainly as discrete, highly mobile organelles undergoing frequent but transient fusion and fission events [47]. Although fusion is observed, many core components of the fusion machinery remain unidentified in plants [30,48,49].

Plant mitochondrial genomes are also unusually large and variable, ranging from ∼208 kb to over 11 mbp, mainly due to non-coding regions, repeats, and introns rather than gene expansion (Table 1 and Figure 1) [50]. Rather than forming stable circular chromosomes, mtDNA exists as a complex mixture of linear and branched molecules [51,52]. Individual mitochondria may carry 80-450 genome copies, but not necessarily a full genome complement, leading to heteroplasmy [53,54]. This incomplete distribution is countered by extensive mitochondrial mobility and fusion, allowing genetic material to mix and thus overall maintain a full complement of the mitochondrial genome [52,55].

Mitochondrial movement relies on the actin cytoskeleton, with myosin motors transporting mitochondria along dense actin networks [48,56]. Prior to cell division, the mitochondrial organelles are stochastically distributed across the cytoplasm such that an even distribution to the daughter cells is achieved.

### Apicomplexa

Apicomplexan mitochondria are highly specialized, differing significantly from typical eukaryotic mitochondria. Their mtDNA is extremely reduced, encoding only three core respiratory genes, *Cox1*, *Cox3*, and *Cytb*, and highly fragmented rRNAs (Table 1 and Figure 1) [57–59]. In *Plasmodium falciparum*, mtDNA exists as tandem arrays of small linear molecules (∼6 kb), while in *T. gondii*, long-read sequencing revealed mitochondrial molecules between 1.1 and 39 kb, assembled from 29 distinct sequence blocks [57,60]. These blocks encode essential respiratory genes in varied configurations, and cryo-electron microscopy has revealed the atomic structure of *T. gondii*’s mitoribosome, demonstrating how its highly fragmented rRNA is integrated into the ribosomal architecture [61,62].

Despite this reduction, mtDNA replication must synchronize with the nuclear cycle. In *P. falciparum*, mitochondrial replication occurs during schizogony, and organelle partitioning happens late in division [63]. In *T. gondii*, the mitochondrion remains largely excluded from daughter cells early on, entering only during late budding stages [64]. In *P. falciparum*, the mitochondrion forms an extensive branched network that divides to ensure each merozoite inherits a mitochondrion.

Strikingly, apicomplexan mitochondria lack canonical fission and fusion machinery [65,66]. No functional homologs of known fission/fusion genes have been identified, suggesting that mitochondrial division is tightly coupled to the cell cycle without spontaneous remodeling. In *T. gondii*, mitochondrial branching and division occur after centrosome duplication and daughter cell scaffolding, relying on parasite-specific cytoskeletal components, including F-actin and TgMyoF [67].

Although ER-mitochondria contact sites play key roles in fungi and animals, similar to many other eukaryotes, no ERMES homologs are found in Apicomplexa [66,68]. This indicates the evolution of unique tethering mechanisms in these parasites. ER-mitochondria contact points have been observed in *P. falciparum*, especially during schizont development, suggesting roles in lipid transfer and mitochondrial maintenance [69,70]. These contacts are enriched with phosphatidylserine synthase and likely mediate the transfer of phosphatidylserine, phosphatidylethanolamine, and phosphatidylcholine from the ER to the mitochondrion [71,72].

## Mitochondria-related organelles

### Mitosomes

#### Giardia

*Giardia intestinalis* is an anaerobic, unicellular protist and an important human parasite [73]. As a member of the Excavata, it has highly reduced cellular structures, including mitochondrion-related organelles (MROs) called mitosomes (Table 1 and Figure 1) [74,75]. Unlike typical mitochondria, *Giardia*’s mitosomes have lost their genome and oxidative phosphorylation machinery. However, they still display hallmarks of mitochondria, including the double membrane and a translocase of the outer and inner mitochondrial membrane complex (TOM/TIM complex) as protein import machinery, as well as the capacity for Fe–S cluster biosynthesis [76,77]. Although these types of structures are also found in other protozoa, including, for example, *Cryptosporidium* spp. and *Entamoeba histolytica*, we focus here on the mitosomes of *Giardia* [78].

Mitosomes are small vesicles (∼40–50 per cell, ∼200 nm in size) dispersed in the cytoplasm, where a distinct subset, the central mitosomes, are positioned between the two nuclei [76,79]. They are physically tethered to the caudal flagella axonemes via a specialized microtubular connector, indicating a key role in inheritance [80]. Unlike mitochondria in opisthokonts, *Giardia* mitosomes do not fuse, resulting in heterogeneous organelle populations over time [79].

Mitosomal division is tightly coupled to mitosis. Rather than continuous fission throughout the cell cycle, mitosomes divide specifically during mitotic phases. Central mitosome division occurs early in prophase, synchronized with basal body segregation and mitotic spindle formation. This precise timing ensures faithful distribution to daughter cells [81]. Inheritance of mitosomes relies on their attachment to the maturing flagellar apparatus. Caudal flagella, representing the oldest and most stable flagella, anchor mitosomes via the microtubular connector [81]. As basal bodies and associated axonemes migrate during mitosis, the tethered mitosomes are mechanically partitioned into daughter cells.

Interestingly, *Giardia*’s mitosomal division appears independent of classical DRPs that mediate mitochondrial fission in other eukaryotes [79]. Although *Giardia* has a DRP homolog, it does not contribute to mitosome division, and the responsible mitosome division machinery remains unknown [79].

Overall, *Giardia* exemplifies a highly unique organelle inheritance system, adapted to life without a mitochondrial genome, mitochondrial fusion, or canonical organelle division machinery.

### Hydrogenosomes

#### Trichomonas vaginalis

Hydrogenosomes in *T. vaginalis* are mitochondrion-related organelles that lack a mitochondrial genome and generate ATP and molecular hydrogen under anaerobic conditions [82]. Typically, spherical or slightly elongated, the hydrogenosomes are enclosed by a double membrane, and their average diameter is about 300 nm, although they can enlarge up to 2 μm under stress (Table 1 and Figure 1) [83]. These organelles replicate only through the proliferation of existing hydrogenosomes via distinct division modes such as fission, partition, or heart-shaped invagination, independent of the cell cycle phase [82]. During asexual division, hydrogenosomes are actively segregated by aligning with cytoskeletal structures like the axostyle and the costa, clustering around the nucleus at mitosis onset, and migrating with the axostyle during karyokinesis [83]. The axostyle is a microtubule-based cytoskeletal rod that extends from the region near the nucleus and basal bodies toward the posterior end, while the costa is a striated fiber running parallel to the flagellum, closely associated with the flagellar basal body and supporting the undulating membrane.

Although the hydrogenosomes of *Trichomonas* lack any mitochondrial DNA, the anaerobic ciliate *Nyctotherus ovalis* retains a mitochondrial genome in its hydrogenosome, which is proposed to be linear and about 40 kbp in length, although the full sequence has not been resolved (Table 1) [82].

**Table 1:** Organellar segregation mechanisms across different organisms

Organism	Organelle	Genomic features	Segregation mechanism	References
Plants/algae	Chloroplast	Mixed structures (120–190 kb); multiple genome copies per nucleoid; anchored to membrane and organized by HU-like proteins	Nucleoid rearrangement during division; possible coupling with cell division; FtsZ ring plus PD rings guide constriction	[4,5,7,9]
Apicomplexa	Apicoplast	Circular, ca. 35 kb in size; single nucleoid with multiple copies of the genome; packaged by HU-like proteins	Single apicoplast per cell; division coordinated with cell cycle; tethered to centrosomes (*T. gondii*) or centriolar plaques (*P. falciparum*); actomyosin-based segregation	[14–20]
Yeast	Mitochondria	Linear concatemers (75–150 kb) with some circular forms; mtDNA packaged into nucleoids (3-4 genomes) by Abf2	Directed, non-random; nucleoids linked to cytoskeleton and ERMES; actin-mediated bud transport	[22,24,25,29]
Mammalian cells	Mitochondria	Circular 16.5 kb mtDNA; compacted into single-genome nucleoids by TFAM	asynchronously throughout the cell cycle; Segregation via frequent fission/fusion, ERMD, and cytoskeletal transport during division	[32,34,39,41,42]
Plants/algae	Mitochondria	Highly variable size (200 kb–11 Mb in plants); mixture of branched molecules and linear concatemers; packaged in multiple copies per nucleoid	Stochastic segregation, genome mixing via frequent fusion events	[47,48,52]
Apicomplexa	Mitochondria	Small linear, tandemly repeated molecules, fragmented and packaged into nucleoids; 6 kb in *P. falciparum*; 1.1 - 39kB in *T. gondii*	Single mitochondrion per cell; division coordinated with cell cycle; closely associated with apicoplast	[57–60]
*Giardia*	Mitosome	No genome	Directed, cell-cycle-coupled inheritance; mitosomes divide during mitosis and are segregated via tethering to caudal flagella and basal bodies	[81]
*Trichomonas vaginalis*	Hydrogenosome	No genome	Active segregation via axostyle and costa; clustering at mitosis onset and migration with the axostyle during karyokinesis	[82,83]
*Nyctotherus ovalis*	Hydrogenosome	proposed linear mitochondrial DNA (∼40 kb)	Unknown	[82]
Kinetoplastids (*Trypanosoma brucei*)	Mitochondria	Complex network of mini- and maxicircles, organized into a single nucleoid and packaged by histone-like KAP proteins	Physical linkage to the basal bodies via the TAC	[84–87]

## The mitochondrial nucleoid—tethered to the IMM, what do we know?

Recently, two non-exclusive models have been proposed that both explain the widely observed regulation of mitochondrial nucleoid positioning [88]. The IMM-dependent trafficking model emphasizes direct tethering of nucleoids to the IMM via proteins like ATAD3A, Twinkle, TFAM, and Mic60, which influence mtDNA integrity, replication, and cristae structure (reviewed in [88]). Conversely, the intercompartment-driven model incorporates broader spatial interactions involving ER-mitochondria contact sites and microtubule-based transport. Some of the same proteins, like ATAD3A in the Twinkle-ATAD3-SAMM50 complex or TFAM in the TFAM-Mic60-Miro-KIF5 axis, mediate inter-organelle communication and active nucleoid movement (reviewed in [88]). According to the models, the functional overlap of these proteins underscores their multifaceted roles in mitochondrial genome maintenance and spatial organization. While very different in their overall organization, both models suggest a direct interaction of the nucleoid with the IMM, often referred to as a tether. However, the nature of this tether remains unknown. While a transmembrane protein like ATAD3A, with domains on either side of the IMM, seems a promising candidate, direct experimental evidence for it to play a role in tethering the nucleoid to the IMM is lacking. Alternatively, the nucleoid might also simply attach to specific lipid microdomains of the inner membrane. In this context, mtDNA molecules have been shown to directly interact with cardiolipin, an IMM-enriched lipid crucial for mtDNA stability and segregation during mitochondrial stress [89]. Moreover, evidence indicates that the mitochondrial helicase Twinkle can transiently bind mtDNA to form a replication platform at cholesterol-rich sites of the IMM, pointing to the importance of lipid composition in organizing and anchoring nucleoids [90].

Taken together, while there is a general consensus that the nucleoid is tethered to the IMM and potentially also to cytoskeletal structures in the cell, experimental evidence for the molecular players is currently lacking, except in one model system where this phenomenon has been worked out in great detail—the tripartite attachment complex (TAC) in *Trypanosoma brucei* [84,87].

## *Trypanosoma brucei* and mitochondrial genome inheritance

**Figure 2:**
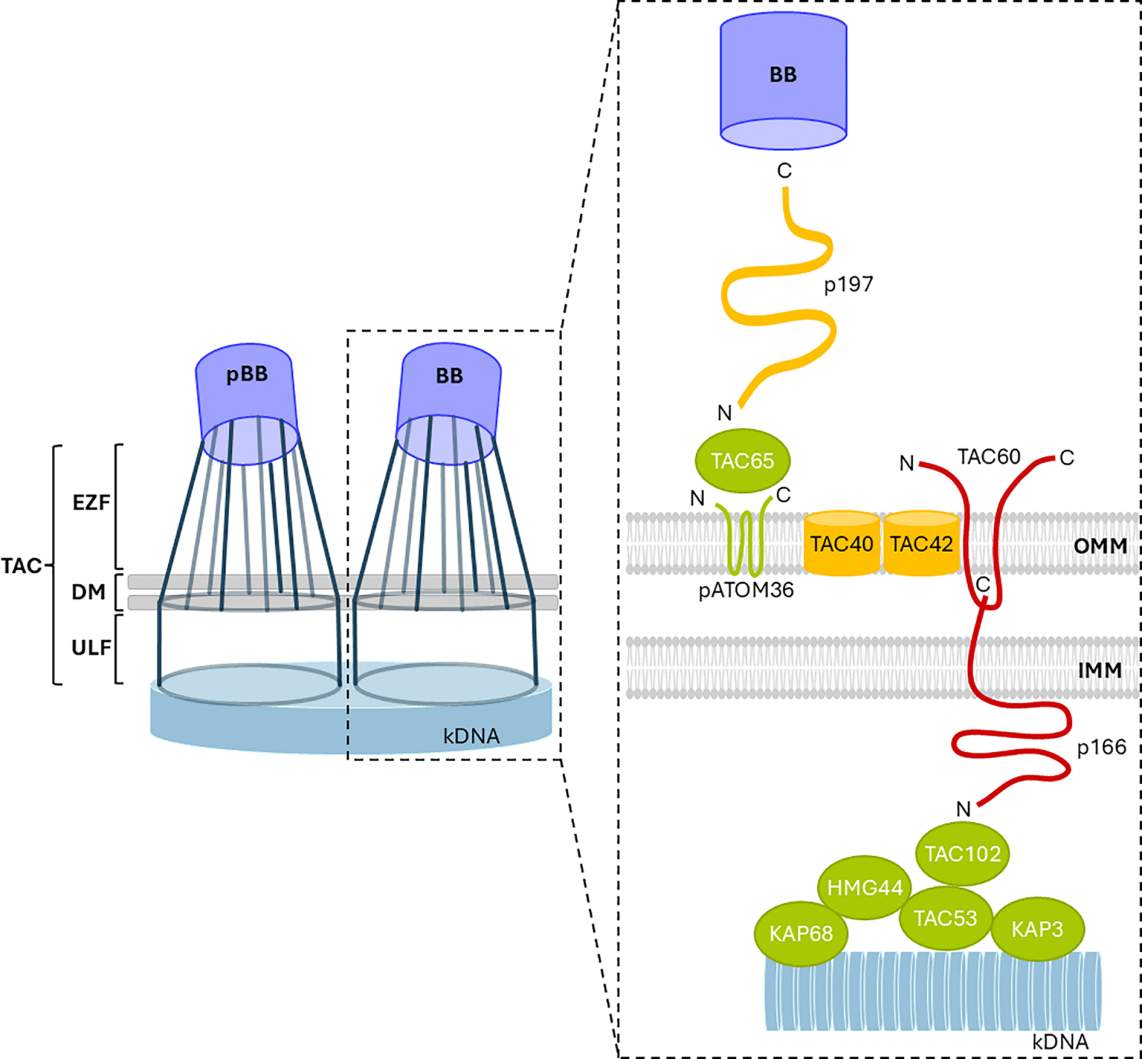
Molecular and structural organization of the tripartite attachment complex The TAC is composed of two tube-like structures, each extending from either the pro-basal body (pBB) or the basal body (BB) to the kDNA. On the left, the three morphological domains of the TAC are illustrated: the exclusion zone filaments (EZF), the differentiated membranes (DM), and the unilateral filaments (ULF). On the right, the proposed molecular organization of the nine known TAC subunits is shown. Additionally, three TAC-associated proteins, HMG44, KAP68, and KAP3, are depicted as potential mediators of the TAC-kDNA linkage. All proteins are color-coded based on their isoelectric point (pI): yellow for neutral, red for acidic, and green for basic pI values.

The protozoan parasite *T. brucei*, the causative agent of human African trypanosomiasis, offers a striking model of organelle genome inheritance. This eukaryote possesses a single flagellum that emerges from the basal body, a single mitochondrion extending the cell’s length, and a unique mitochondrial nucleoid: the kDNA (Table 1, Figures 1 and 2) [84]. The kDNA is a disc-shaped network composed of thousands of interlocked DNA molecules, including two types: maxicircles and minicircles. Maxicircles encode 18 mRNAs and two rRNAs, while minicircles produce guide RNAs essential for maxicircle transcript editing [91].

Because *T. brucei* has only one kDNA network, faithful segregation during cell division is critical. This is achieved by tightly coupling kDNA replication and segregation to the cell cycle. As basal bodies duplicate and migrate apart, they pull the duplicated kDNA into two masses, each destined for a daughter cell. This mechanical linkage is mediated by the TAC (Table 2 and Figure 2) [86,87].

**Table 2:** The nine core TAC components

Protein	Gene ID	Isoelectric point	Molecular weight [kDa]	Information	References
p197	Tb927.10.15750	7.46	880	Most basal-body-proximal TAC protein; links basal body to TAC65 at OMM; central domain (∼35 spectrin-like repeats) forms flexible coiled-coil spanning EZF length	[93–95]
TAC65	Tb927.5.830	10.08	65	Peripheral OMM protein anchoring p197 to mitochondrial surface via pATOM36; essential for OMM TAC subcomplex stability	[94,96]
pATOM36	Tb927.7.5700	10.66	36	Integral OMM protein with dual roles: links TAC65 to OMM and functions in OMM protein biogenesis	[96–98]
TAC40	Tb927.4.1610	7.02	40	Integral β-barrel proteins in DMs: TAC40 (VDAC-like) and kinetoplastid-specific TAC42 form scaffold; interact with TAC60 to bridge outer and inner membranes	[99,100]
TAC42	Tb927.7.3060	6.59	42		
TAC60	Tb927.7.1400	4.95	60	OMM protein with two TMDs; intermembrane space (IMS) loop binds p166, cytosolic ends interact with TAC40 and TAC42	[99,101,102]
p166	Tb927.11.3290	5.13	166	IMM protein with matrix-facing coiled-coil and short IMS tail binding TAC60; links OMM components to matrix TAC proteins	[101–103]
TAC102	Tb927.7.2390	9.41	102	Matrix ULF protein with high pI; marks transition from membrane-bound TAC to DNA-associated region	[104,105]
TAC53	Tb927.2.6100	10.77	53	Recently identified protein closer to kDNA than TAC102; likely DNA-binding (AT-hooks, SPKK motifs) but lacks direct biochemical confirmation	[106]

### The tripartite attachment complex

The TAC is a filamentous structure physically connecting the kDNA disc to the flagellar basal body, spanning both mitochondrial membranes (Table 2 and Figure 2) [87]. As the basal bodies segregate, the TAC ensures that replicated kDNA is pulled apart and accurately partitioned. Electron microscopy revealed three regions of the TAC:

EZF: Extend from the basal bodies to the outer mitochondrial membrane (OMM), traversing a ribosome-free cytoplasmic region.
DM: Specialized zones where the TAC crosses the outer and inner membranes, showing detergent resistance and altered protein composition.
ULF: Matrix-side filaments connecting the inner membrane to the kDNA, subdivided into a DNA-free, acidic region and a DNA-proximal, basic region.

To classify a protein as a core TAC subunit, three criteria must be met: depletion causes kDNA missegregation; it is dispensable in engineered bloodstream forms surviving without kDNA (yL262P line); and it localizes between the basal body and the kDNA. An exception is pATOM36, which has dual roles inside and outside the TAC [84,85].

The TAC assembles hierarchically from the basal body toward the kDNA [92]. Disruption of upstream (basal-body-proximal) components destabilizes downstream elements, but not vice versa. Currently, nine TAC proteins are identified.

## Common principles of organelle inheritance

Across eukaryotic life, several fundamental strategies ensure the faithful inheritance of organellar genomes. A common feature is the tight coupling of organelle genome replication and segregation with the cell cycle, as seen in *T. brucei*, *T. gondii*, and *S. cerevisiae*, where timing is synchronized with nuclear events to prevent missegregation [17,22,84]. Another widespread strategy is the use of physical tethering mechanisms to anchor organelles or their genomes to key cellular structures. In yeast, mitochondria are anchored via their outer membrane to the ER through ERMES to position replicating nucleoids at division sites [29]. In *T. gondii*, the apicoplast is connected to centrosomes, allowing its faithful inheritance as the cell divides [17]. Similarly, in *T. brucei*, the kDNA is physically linked to the basal bodies via the TAC, which pulls the replicated genome into daughter cells [85].

Cytoskeletal elements, particularly actin, myosin, and microtubules, also contribute to organelle positioning and transport. Myosins facilitate directional movement in yeast and apicomplexans, while microtubular connections enable mitosome inheritance in *Giardia* and hydrogenosomes in *Trichomonas* [18,24,81,83]. Division of mitochondria and apicoplasts is often mediated by DRPs, or in chloroplasts, by the bacterial-derived FtsZ ring [3,44,107,108]. Nucleoid organization is another conserved theme. Across species, organellar DNA is tightly packaged with basic proteins, such as TFAM in mammals, KAPs in trypanosomes, and Abf2 in yeast [25,39,86].

While there is seemingly a large diversity in the structures involved in organelle and, more specifically, organellar DNA segregation, the only well-characterized machinery remains the TAC in *T. brucei.* The TAC is a highly specialized mitochondrial genome segregation apparatus that is conserved across Kinetoplastea but likely architecturally diversified. Major unresolved questions concern (i) how TAC architecture varies among kinetoplastids with less catenated mitochondrial genomes, (ii) how such a *trans*-membrane megastructure assembles and is regulated during the cell cycle, and (iii) how extraordinarily large components such as p197 are synthesized, targeted, and spatially organized. Comparative genomics and cell biological studies suggest conservation of ‘core’ elements but likely substantial divergence in DNA-proximal modules. The coming years will require integration of evolutionary cell biology, high-resolution structural methods, *in situ* translation assays, and comparative analysis beyond *T. brucei*.

## Perspectives

- The faithful segregation of organellar genomes represents a fundamental aspect of eukaryotic cell biology with direct implications for cellular survival, organismal health, and disease. Unlike nuclear DNA, whose segregation is mediated by the mitotic spindle, organelle genome inheritance relies on a diversity of strategies that vary across taxa, including stochastic processes, cytoskeletal transport, and membrane tethering. This diversity highlights both the evolutionary plasticity of organelle biology and the absence of a universal mechanism for genome segregation.
- This review highlights the complex and diverse strategies utilized by various organisms to address the fundamental biological challenge of organellar genome distribution. These mechanisms include stochastic and coordinated processes involving active transport along the cytoskeleton and specialized tethering to organellar membranes. Despite the mechanistic variability across species, several conserved principles emerge. These include the spatial organization of nucleoids, their anchoring to membrane structures, and the involvement of cytoskeletal elements in facilitating genome positioning and inheritance.
- Beyond their biomedical importance, studies across systems reveal unifying biological principles. Organelle genome replication and segregation are tightly coupled to the cell cycle, often relying on physical tethering to key cellular structures and conserved cytoskeletal or dynamin-related proteins. Together, this body of work illustrates how eukaryotes have evolved diverse yet convergent solutions to the universal challenge of organelle genome inheritance, offering both mechanistic insights and opportunities for translational advances.

## Data Availability

All data available and included in the manuscript

## Open Access

Open access for this article is provided via our Subscribe to Open programme, made possible through the support of our subscribing institutions. Learn more here: https://portlandpress.com/pages/open_access_options_and_prices#conditional

## Abbreviations

BB: basal body
DM: differentiated membranes
DRPs: dynamin-related proteins
ERMD: ER-associated mitochondrial division
EZF: exclusion zone filaments
IMM: inner mitochondrial membrane
IMS: intermembrane space
kb: kilobases
KAPs: kinetoplast-associated proteins
kDNA: kinetoplast DNA
MROs: mitochondrion-related organelles
OMM: outer mitochondrial membrane
PD: plastid-dividing
pBB: pro-basal body
TAC: tripartite attachment complex
ULF: unilateral filaments
